# Safety of Live Attenuated MMR, Varicella, and Yellow Fever Vaccination in Patients with Inflammatory Bowel Disease Receiving Biologic and Targeted Synthetic Therapy: A Propensity-Score-Matched Analysis

**DOI:** 10.3390/vaccines14060474

**Published:** 2026-05-26

**Authors:** Niven Wang, Abdelrahman Yousef, Kevin Nguyen, Timothy Mok, Mahmoud Yousef, Ahmed Telbany, Abu Baker Sheikh, Christopher Chang, Swathi Paleti

**Affiliations:** 1 Internal Medicine Department, The University of New Mexico Health Sciences Center, University of New Mexico, Albuquerque, NM 87131, USA; nlwang@salud.unm.edu (N.W.); abyousef@salud.unm.edu (A.Y.); kknguyen@salud.unm.edu (K.N.); absheikh@salud.unm.edu (A.B.S.); 2 The University of Texas Health Science Center at Houston, Houston, TX 77054, USA; timothy.mok@uth.tmc.edu; 3 General Medicine Department, SUNY Upstate Medical University, Syracuse, NY 13210, USA; yousefma@upstate.edu; 4 Division of Gastroenterology and Hepatology, The University of New Mexico Health Sciences Center, University of New Mexico, Albuquerque, NM 87131, USA; aeltelbany@salud.unm.edu (A.T.); cchang1@salud.unm.edu (C.C.); 5 Gastroenterology Department, Raymond G. Murphy Veterans Affairs Medical Center, Albuquerque, NM 87108, USA

**Keywords:** inflammatory bowel disease, live attenuated vaccines, immunomodulatory therapy, infliximab, vedolizumab

## Abstract

Introduction: Live attenuated vaccines (LAVs) are generally avoided in patients with inflammatory bowel disease (IBD) receiving immunomodulatory therapy due to concerns about infection risk. However, real-world data evaluating their safety in this population remain limited. We aimed to assess adverse outcomes following LAV administration in IBD patients treated with biologic agents. Methods: We conducted a retrospective cohort study using the TriNetX multi-institutional database. Adults with IBD receiving immunomodulatory therapy were categorized into two cohorts: those who received an LAV and those who did not. Biologic therapies included tumor necrosis factor inhibitors (infliximab, and adalimumab), integrin antagonists (vedolizumab), interleukin (IL)-12/23 inhibitors (ustekinumab), IL-23 inhibitors (risankizumab, and guselkumab), and Janus kinase inhibitors (tofacitinib, and upadacitinib). LAVs included measles–mumps–rubella (MMR), varicella (Varivax), and yellow fever vaccines. Propensity score matching was performed based on age, sex, IBD subtype (Crohn’s disease vs. ulcerative colitis), and biologic class. Patients with outcomes prior to the risk window were excluded. Adverse outcomes within six months included hospitalization, emergency department (ED) visits, fever, rash, and encephalitis. Results: A total of 672 patients were included in each propensity-score-matched cohort. Live attenuated vaccine (LAV) administration was not associated with significantly increased adverse outcomes compared with no LAV exposure during the six-month follow-up period. Hospitalization occurred in 14.9% versus 15.3% of patients, respectively (risk ratio [RR] 0.97; 95% confidence interval [CI] 0.75–1.25; *p* = 0.819), while emergency department visits occurred in 12.6% vs 11.3% (RR 1.12; 95% CI 0.84–1.50; *p* = 0.450). There were no significant differences in fever (3.6% vs. 3.3%; RR 1.09; 95% CI 0.62–1.93; *p* = 0.764) or rash (4.0% vs. 2.7%; RR 1.50; 95% CI 0.83–2.70; *p* = 0.172). No cases of measles, mumps, rubella, varicella, yellow fever, or encephalitis were identified in either cohort during follow-up. Conclusions: LAVs were not associated with an increased risk of adverse outcomes within one day to six months among IBD patients receiving immunomodulatory therapy. These real-world findings suggest comparable short-term outcomes between the cohorts of patients with IBD receiving biologic or targeted synthetic therapy who met the predefined eligibility criteria including age ≥ 18 years, and vaccination occurring between two weeks and six months after biologic initiation regarding LAV use in patients with IBD receiving biologic agents.

## 1. Introduction

Inflammatory bowel disease (IBD) represents a spectrum of chronic, relapsing gastrointestinal autoimmune disorders, primarily categorized as Crohn’s disease (CD) or ulcerative colitis (UC). The pathogenesis of IBD is characterized by chronic, disproportionate mucosal inflammation, widely hypothesized to result from a dysregulated immune response directed against components of the intestinal microbiota and environmental antigens [1]. This exaggerated immune activation drives epithelial barrier disruption, culminating in hallmark clinical symptoms such as abdominal pain, gastrointestinal bleeding, and deep ulcerations. Pathologically, UC is defined by continuous inflammation restricted to the colon, originating at the rectum, while CD is distinguished by transmural, discontinuous lesions (skip lesions) that can affect any segment of the alimentary tract from the oral cavity to the anus [1]. Epidemiologically, IBD exhibits a slight female predominance and displays a bimodal distribution of diagnosis, with the highest incidence observed in young adults aged 15 to 30 years and a secondary peak occurring in the population over 60 years of age [1,2,3].

IBD can be treated in numerous ways, which depend on the severity of the disease. Patients can be initially treated with aminosalicylates with or without the use of corticosteroids [3]. The use of corticosteroids can be added if there are acute flare-ups. In patients who have multiple flares, newer medications like immune modulator therapy are indicated. Infliximab, a tumor necrosis factor inhibitor (anti-TNF), was approved for autoimmune disease in 1998 [3]. This selectively inhibited TNF alpha, which blocked the inflammation cascade (IL-1 and IL-6). Modern IBD therapeutics variably modulate host immunity: TNF inhibitors dampen macrophage and Th1 signaling; anti-integrins restrict gut homing; IL-12/23 and IL-23 inhibitors target the Th1 and Th17 axes; and JAK inhibitors attenuate cytokine signaling across multiple pathways [3,4,5,6,7].

Live attenuated vaccines (LAVs) contain replication-competent pathogens attenuated to elicit robust cellular and humoral immunity [8]. In immunocompetent hosts, these vaccines are highly effective and safe. However, in patients with impaired T-cell-mediated immunity, LAVs carry a theoretical risk of uncontrolled replication and disseminated vaccine-strain infection [9]. This risk is supported primarily by rare but severe cases observed in individuals with congenital immunodeficiency syndromes and profound T-cell dysfunction. Emerging evidence highlights additional layers of immune dysregulation in IBD, including the role of microRNAs in modulating inflammatory pathways, which may influence both the efficacy and safety of immunomodulatory therapies and their interactions with live vaccines. As a result, major professional societies—including the Centers for Disease Control and Prevention, Infectious Diseases Society of America, American College of Gastroenterology, and Canadian Association of Gastroenterology (CDC/IDSA/ACG/CAG)—recommend completing the indicated LAVs at least four weeks before initiating high-level immunosuppression and generally avoiding LAVs thereafter, except in select case-by-case circumstances such as unavoidable travel-related yellow fever vaccination [8,10,11,12,13,14,15]. Despite the theoretical risks, large-scale real-world safety data on live attenuated vaccine (LAV) administration in patients receiving biologic or targeted synthetic therapy for IBD remains conspicuously absent. The existing evidence is largely confined to small case series, registry data, and extrapolations from other immunosuppressed populations.

Adults with IBD remain significantly under-vaccinated compared with the general population, despite their increased susceptibility to vaccine-preventable infections [1,15]. Multiple factors contribute to this persistent gap, including the fragmented coordination of preventive care, uncertainty regarding the timing of vaccine administration relative to immunosuppression, and current guidelines that state LAVs are contraindicated once biologic treatment is initiated [8,13]. Given that patients with IBD are often dependent on their gastroenterologists for the close management of their symptoms, a multidisciplinary approach with the patient’s primary care team is pertinent for increasing vaccine administration for these patients. Consequently, major societies (CDC/IDSA/ACG/CAG) recommend completing the indicated LAVs before high-level immunosuppression and generally avoiding them thereafter, except with case-by-case exceptions such as travel-related yellow fever [10]. However, the diverse mechanisms of modern immunologic treatments (TNF inhibitors, IL12/23 inhibitors, and JAK inhibitors) suggest that not all pose the same risk; a recent study investigating LAV safety in patients receiving the gut-selective anti-integrin agent, vedolizumab (VDZ), demonstrated promising safety signals, with no cases of vaccine-induced infection observed during a median 121-week follow-up and high seroconversion rates (91.9%) [16].

In real-world practice, clinicians frequently encounter scenarios in which LAVs cannot be completed prior to biologic initiation, such as the late diagnosis of non-immunity, outbreak exposure, or imminent travel to endemic regions. The lack of large-scale real-world safety data leaves clinicians and patients navigating these decisions with limited evidence.

To address this critical knowledge gap, we conducted a multicenter, propensity-score-matched cohort study using a national federated electronic health record network to evaluate the safety of exposure to Measles Mumps and Rublla (MMR), Varicella, and Yellow Fever vaccines after immunomodulatory therapy in adults with IBD. MMR, varicella, and yellow fever vaccines were selected because they are the LAVs most commonly encountered in clinical practice within the United States for adults with IBD in travel, occupational, or catch-up vaccination contexts, and because they carry the highest theoretical risk of disseminated vaccine-strain illness in immunosuppressed hosts. We assessed clinically meaningful outcomes across short-term, post-biologic, and medium-term windows using both absolute risk and time-to-event analyses.

## 2. Methods

### 2.1. Study Design and Data Source

We performed a retrospective, propensity-score-matched cohort study using the TriNetX Research Network. Data were obtained from the TriNetX US Collaborative Network, a federated real-world data platform encompassing electronic health records (EHRs) from over 60 healthcare organizations across the United States, including academic medical centers, community hospitals, and specialty practices. TriNetX data are de-identified in accordance with HIPAA Safe Harbor standards. The network returns only aggregate counts, measures of association, and prebuilt survival outputs; individual charts are not accessible. All cohort creation, covariate selection, propensity matching, and statistical outputs were generated within the browser-based TriNetX Live analytics environment.

The study period spanned available data within the TriNetX network (2000–2024). Zoster vaccination (Zostavax) was not included due to inconsistent capture and transition to recombinant vaccines during the study period.

### 2.2. Study Population

We identified adults (≥18 years) with IBD and exposure to a biologic or targeted small-molecule immunomodulator. IBD was defined using ICD-10 codes for Crohn’s disease and ulcerative colitis. Biologic/targeted therapy classes included TNF inhibitors (infliximab, and adalimumab), anti-integrin (vedolizumab), IL-12/23 inhibitor (ustekinumab), IL-23 inhibitors (risankizumab, and guselkumab), and JAK inhibitors (tofacitinib, and upadacitinib). Drug exposures were ascertained from RxNorm medication/administration tables and, for infused/injected therapies, HCPCS J-codes; oral agents were identified by RxNorm/NDC.

### 2.3. Code Sets (ICD-10, CVX/CPT, and RxNorm/HCPCS)

Patients with inflammatory bowel disease (IBD) were identified using ICD-10 diagnostic codes for Crohn’s disease (K50) and ulcerative colitis (K51). Exposure to biologic and targeted therapies was identified using RxNorm medication codes for infliximab (191831), vedolizumab (1538097), ustekinumab (847083), adalimumab (327361), upadacitinib (2196092), risankizumab (2166040), guselkumab (1928588), and tofacitinib (1357536).

Live attenuated vaccine (LAV) exposure was identified using vaccine and medication code sets for measles–mumps–rubella (MMR; CVX 3), yellow fever vaccine (CVX 184), and live attenuated varicella-zoster vaccine (RxNorm 1292422). Patients in the exposure cohort were required to receive at least one of these vaccines within 14 days to 6 months following biologic or targeted therapy initiation, while patients in the comparator cohort were excluded if they had documentation of any live attenuated vaccine exposure.

Additional baseline comorbidity variables used for propensity score matching included diabetes mellitus (E08–E13), human immunodeficiency virus disease (B20), common variable immunodeficiency with predominant immunoregulatory T-cell disorders (D83.1), other specified immunodeficiencies (D84.8), defects in the complement system (D84.1), leukemia unspecified (C95.9), and malignant neoplasm unspecified.

Clinical outcomes were defined using ICD-10 and standardized encounter coding definitions within the TriNetX platform. Inpatient visits were identified using HL7 visit type code IMP (inpatient encounter), while emergency department visits were identified using HL7 visit type code EMER. Fever was defined using ICD-10 code R50.9 (fever, unspecified), rash using ICD-10 code R21 (rash and other nonspecific skin eruption), and encephalitis using ICD-10 code G04 (encephalitis, myelitis, and encephalomyelitis). Vaccine-related infectious outcomes included measles (B05), mumps (B26), rubella (B06), varicella/chickenpox (B01), and yellow fever (A95).

### 2.4. Exposure Definitions and Index Dates

The exposure cohort consisted of patients with inflammatory bowel disease (IBD) receiving biologic or targeted therapy who subsequently received a live attenuated vaccine (LAV), including measles–mumps–rubella (MMR), yellow fever vaccine, or live attenuated varicella vaccine. Eligible biologic and targeted therapies included infliximab, adalimumab, vedolizumab, ustekinumab, risankizumab, guselkumab, tofacitinib, and upadacitinib. Patients in the comparator cohort were required to have exposure to biologic or targeted therapy without documented receipt of a live attenuated vaccine during the study period.

Exposure to live attenuated vaccination was defined by receipt of at least one qualifying vaccine occurring after initiation of biologic or targeted therapy. The exposure window of 2 weeks to 6 months after biologic initiation was selected to balance clinical plausibility with data availability; however, we acknowledge that patients vaccinated early in this window may not have achieved full immunosuppression, potentially introducing misclassification bias. Individual-level verification of immunosuppressive status was not possible in this de-identified dataset. The unexposed cohort comprised biologic-treated IBD patients who have never been exposed to LAVs after starting biologics. Immunizations were captured from EHR immunization tables using vaccine CVX codes and corroborating CPT codes when available. Index event was the date of vaccine administration in Cohort 1 and the date of biologic administration in Cohort 2 within the last 60 days.

### 2.5. Analytic Windows

We prespecified two windows: (1) primary 1-day-to-6-month analysis; and (2) post-biologic subanalysis restricting outcomes within 1 day to 3 months. The shorter window was included to better capture adverse events occurring more directly after receiving LAV. However, there is no definitive method to ensure direct correlation.

### 2.6. Outcomes

Primary outcomes were any inpatient admission, and any emergency department visit during each analysis window, derived from encounter-type metadata. Secondary diagnosis-based outcomes included fever, rash, encephalitis, varicella, and vaccine-virus diagnoses (measles, mumps, rubella, and yellow fever). For patients who experienced an outcome, we also summarized the number of repeated instances (e.g., number of admissions).

### 2.7. Covariates and Matching

We captured age, sex, race/ethnicity, IBD subtype, biologic/targeted class, selected comorbidities, and concomitant immunosuppressants. We then performed 1:1 propensity score matching with covariates age, sex, race/ethnicity, IBD subtype, biologic/targeted class, and concomitant immunosuppressants. Matching used greedy nearest-neighbor with a 0.1 SD caliper without replacement. Balance was evaluated with standardized mean differences (SMD); SMD < 0.10 was prespecified as acceptable.

Biologic/targeted therapy class and concomitant immunosuppressive medications (e.g., corticosteroids, and thiopurines) were included in propensity score matching; however, detailed distributions of these therapies are not fully captured in Table 1 and represent an important limitation given variability in systemic immunosuppressive effect across agents (e.g., vedolizumab vs. TNF inhibitors).

### 2.8. Statistical Analysis

All analyses were executed within TriNetX platform. Following propensity score matching, outcome comparisons between cohorts were conducted using TriNetX Compare Outcomes analytics. We estimated absolute risks, risk differences (RDs), risk ratios (RRs), odds ratios (ORs), and 95% confidence intervals for matched cohorts. A number of event analyses was performed to evaluate the frequency of outcome-related events occurring within the study time window. This analysis quantified how many times a given outcome occurred among patients who experienced at least one event during follow-up. Outcomes were evaluated from 1 day to 180 days following the index event.

## 3. Results

### 3.1. Baseline Demographics

Prior to propensity score matching, the biologics with the live attenuated vaccine (LAV) cohort included 672 patients, while the biologics without the LAV cohort included 144,165 patients. After 1:1 propensity score matching, 672 patients remained in each cohort.

Following matching, the baseline demographic characteristics were well-balanced between cohorts. The mean age at the index was 38.8 ± 17.6 years in the biologics with the LAV cohort and 39.0 ± 17.8 years in the biologics without the LAV cohort. Female patients comprised 73.7% and 73.8% of the cohorts, respectively, while male patients accounted for 26.3% and 26.2%.

With respect to race and ethnicity, White patients represented 76.2% of the LAV cohort and 76.6% of the non-LAV cohort. Black or African American patients accounted for 13.5% and 14.0% of patients, respectively, while Asian patients comprised approximately 4% of both cohorts. The Hispanic or Latino ethnicity was present in 5.5% of the LAV cohort and 5.4% of the non-LAV cohort.

**Figure 1:**
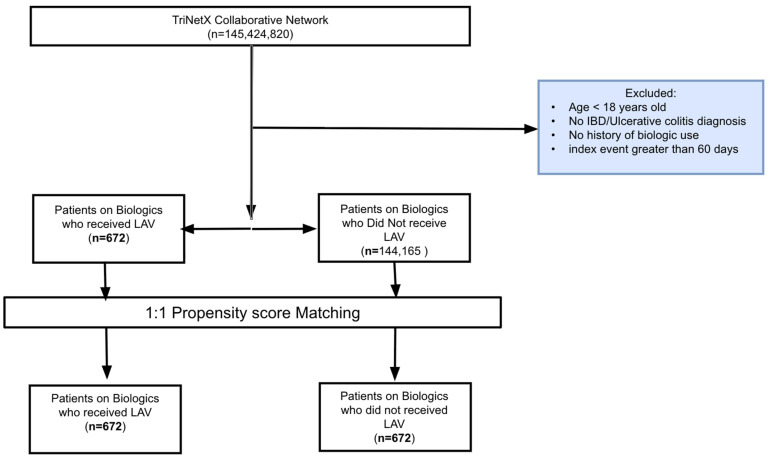
Flow diagram of patient selection. This flowchart illustrates patient selection from the TriNetX Research Network. Patients were identified based on a diagnosis of inflammatory bowel disease and exposure to biologic or targeted synthetic therapy. Exclusion criteria included age younger than 18 years and patients who received live attenuated vaccination prior to initiation of biologic or targeted therapy. After propensity score matching, the final study cohorts included 672 patients in each group.

Disease characteristics were also comparable following matching. Crohn’s disease was present in 58.9% of patients in both cohorts, while ulcerative colitis was present in 28.9% of the LAV cohort and 29.3% of the non-LAV cohort. Rates of diabetes mellitus, HIV infection, and other immunodeficiency disorders were low and generally similar between cohorts after matching. Overall, the propensity score matching achieved the appropriate balance across the measured demographic and clinical variables (Figure 1, and Table 1).

### 3.2. Primary 1 to 6 Month Outcomes

Following 1:1 propensity score matching, 672 patients were included in each cohort. Outcomes were assessed from 1 day to 180 days following the index event.

Hospitalization occurred in 14.9% of patients with inflammatory bowel disease (IBD) receiving biologic or targeted therapy who also received a live attenuated vaccine (LAV), compared with 15.3% of patients receiving biologic or targeted therapy without LAV exposure (risk ratio [RR] 0.97, 95% CI 0.75–1.25; *p* = 0.819). Emergency department visits occurred in 12.6% versus 11.3% of patients, respectively (RR 1.12, 95% CI 0.84–1.50; *p* = 0.450).

Minor adverse events were infrequent and comparable between cohorts. Fever occurred in 3.6% of vaccinated patients compared with 3.3% of unvaccinated patients (RR 1.09, 95% CI 0.62–1.93; *p* = 0.764). Rash occurred in 4.0% of patients receiving LAVs compared with 2.7% of patients without LAV exposure (RR 1.50, 95% CI 0.83–2.70; *p* = 0.172).

Importantly, there were no recorded cases of encephalitis, measles, mumps, rubella, varicella, or yellow fever in either cohort during the six-month follow-up period.

**Figure 2:**
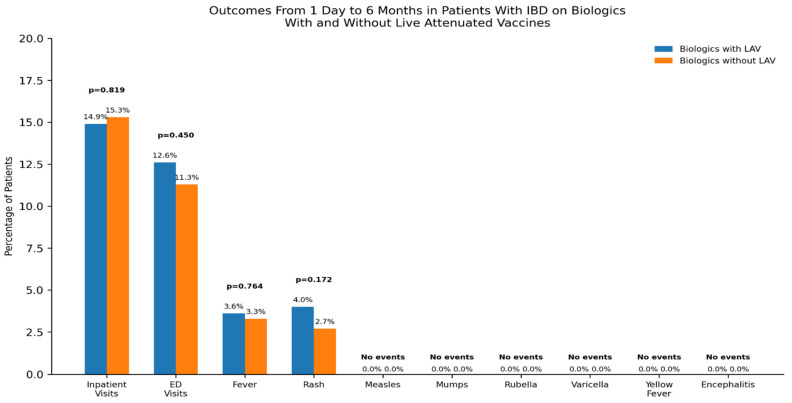
1-day to 6-month outcomes: live attenuated vaccine + biologic vs. no live attenuated vaccine. A bar plot showing major and minor complications.

Overall, no statistically significant differences in hospitalization, emergency department utilization, or vaccine-associated infectious complications were observed between patients receiving biologic or targeted therapy with LAV exposure and those without LAV exposure during intermediate-term follow-up (Figure 2 and Table 2).

The frequency of outcome-related events was additionally evaluated using the TriNetX number of instances analysis. Among patients who experienced hospitalization, the mean number of inpatient encounters was similar between cohorts, with a mean of 2.97 ± 4.10 inpatient visits in the biologics with the live attenuated vaccine (LAV) cohort compared with 2.94 ± 3.10 inpatient visits in the biologics without the LAV cohort (*p* = 0.956). The median number of inpatient encounters was 2 in both groups.

Among patients with emergency department utilization, the biologics with the LAV cohort demonstrated a lower mean number of emergency department visits compared with the non-LAV cohort (1.72 ± 1.08 vs. 2.45 ± 3.08 visits, respectively; *p* = 0.042), although the overall proportion of patients with emergency department visits was not significantly different between groups. The median number of emergency department visits was 1 in both cohorts.

For fever-related events, the mean number of occurrences was comparable between cohorts, with a mean of 1.79 ± 2.45 events in the LAV cohort and 2.09 ± 2.27 events in the non-LAV cohort (*p* = 0.670). The median number of fever-related events was 1 in both groups.

No instances of measles, mumps, rubella, varicella, or yellow fever were identified in either cohort during the six-month follow-up period, and, therefore, no frequency analyses could be performed for these outcomes.

### 3.3. Post-Biologic Subanalysis (1 Day–3 Months After Biologic Start)

After propensity score matching, 672 patients were included in each cohort. Cohort 1 consisted of patients with inflammatory bowel disease (IBD) receiving biologic or targeted therapy who also received a live attenuated vaccine (LAV), while Cohort 2 included patients receiving biologic or targeted therapy without LAV exposure. Outcomes were assessed from 1 day to 90 days following the index event.

Hospitalization occurred in 70 of 672 patients (10.4%) receiving biologics with LAV exposure compared with 68 of 672 patients (10.1%) receiving biologics without LAV exposure (risk ratio [RR] 1.03, 95% CI 0.75–1.41; *p* = 0.857). Emergency department visits occurred in 60 of 672 patients (8.9%) in the LAV cohort versus 47 of 672 patients (7.0%) in the non-LAV cohort (RR 1.28, 95% CI 0.89–1.84; *p* = 0.190). Fever occurred in 14 of 672 patients (2.1%) in both cohorts (RR 1.00, 95% CI 0.48–2.08; *p* = 1.000), demonstrating no difference in risk between groups.

No cases of measles, mumps, rubella, varicella, or yellow fever were identified in either cohort during the 1-day to 3-month follow-up period. Aditionally, rash and encephalitis outcomes were excluded from this analysis because insufficient event data were available to generate a reliable outcome analysis within the selected time window.

Overall, no statistically significant differences in hospitalization, emergency department utilization, fever, or vaccine-related infectious complications were observed between patients receiving biologics with LAV exposure and those without LAV exposure during short-term follow-up.

A number of instances analysis was performed to evaluate the frequency of outcome-related events among patients who experienced each outcome during the 1-day to 3-month follow-up period.

Among patients with inpatient encounters, the mean number of inpatient visits was 2.04 ± 2.30 in the biologics with the LAV cohort compared with 2.82 ± 2.90 in the biologics without the LAV cohort, with median values of 1 and 2 encounters, respectively (*p* = 0.082). For emergency department utilization, patients receiving biologics with LAV exposure had a mean of 1.52 ± 0.91 emergency department visits compared with 2.17 ± 2.38 visits in patients without LAV exposure. The median number of visits was 1 encounter in both cohorts (*p* = 0.053).

**Figure 3:**
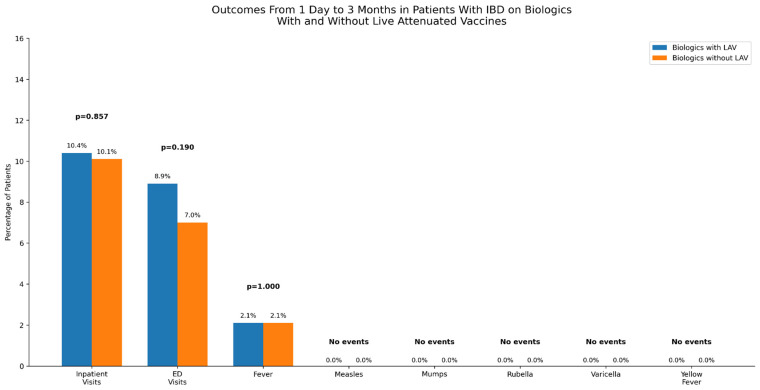
Short-window outcomes (1 day to 3 months). Not enough data was available for measles, varicella, pneumonia, or encephalitis outcomes.

Among patients with fever, the mean number of fever-related instances was 1.93 ± 2.65 in the LAV cohort and 2.36 ± 2.65 in the non-LAV cohort, with a median of 1 fever-related encounter in both groups (*p* = 0.672). No measurable instances of measles, mumps, rubella, varicella, or yellow fever were identified in either cohort during the study period. (Figure 3 and Table 3).

## 4. Discussion

In this multicenter propensity-score-matched cohort of adults with inflammatory bowel disease receiving biologic or targeted therapy, receipt of a live attenuated vaccine (LAV) in proximity to biologic treatment was not associated with significantly increased short-term risks of hospitalization, emergency department utilization, fever, or rash. Across both the 90-day and 6-month analyses, the rates of inpatient and emergency department encounters were comparable between vaccinated and unvaccinated cohorts, with no statistically significant differences observed in major clinical outcomes. Minor adverse events, including fever and rash, were infrequent and similarly distributed between groups. No cases of measles, mumps, rubella, varicella, or yellow fever were identified during follow-up in either cohort. However, given the rarity of these outcomes and limited sample size, the absence of observed events should not be interpreted as definitive evidence of safety. Rare-event analyses remain underpowered, and clinically meaningful risk cannot be excluded. Although some outcomes demonstrated numerically higher event rates in the vaccinated cohort, confidence intervals were wide and crossed unity, limiting definitive interpretation. Overall, these findings did not identify a measurable short-term safety signal associated with live attenuated vaccine exposure among patients receiving biologic or targeted therapy.

The repeat-event analysis demonstrated generally comparable rates of recurrent healthcare utilization between cohorts; however, notable differences were observed in emergency department utilization during the six-month analysis window. Among patients who experienced emergency department visits, vaccinated patients had fewer recurrent ED encounters compared with unvaccinated patients (mean 1.72 ± 1.08 vs. 2.45 ± 3.08 visits; *p* = 0.042). Although the overall risk of experiencing at least one ED visit was not significantly different between groups, this finding suggests that vaccinated patients who required emergency care had fewer repeat ED presentations during follow-up. In contrast, the mean number of inpatient encounters and fever-related events did not significantly differ between cohorts. Importantly, recurrent vaccine-associated infectious complications, including measles, varicella, yellow fever, and encephalitis, were not observed. While the reduced frequency of repeat ED utilization among vaccinated patients is notable, interpretation should remain cautious given the exploratory nature of repeat-event analyses and the relatively small number of patients experiencing recurrent events.

These findings are consistent with the existing literature evaluating the safety of LAVs in immunosuppressed populations. A systematic review by Lee YJ et al. [17] evaluated the available studies of immunosuppressed patients with IBD receiving live attenuated vaccines and found that serious vaccine-related adverse events were rare, with most included studies reporting no cases of disseminated infection or severe complications. Similarly, Pöyhönen L et al. [9] examined life-threatening infections following LAV administration, demonstrating that severe complications are predominantly confined to patients with profound inborn errors of immunity, rather than those with iatrogenic immunosuppression such as IBD therapy. In a focused review, Kamei K [18] assessed LAV use in patients receiving immunosuppressive agents and concluded that, while caution is warranted, adverse outcomes are uncommon when patients are carefully selected and monitored. More recently, Shiga H et al. [16] specifically evaluated patients with IBD receiving vedolizumab who underwent LAV administration, reporting no serious adverse events or vaccine-strain infections, further supporting the relative safety of LAVs in selected patients on targeted therapy. Although this study examined live vaccine safety specifically in the context of anti-integrin therapy, which acts primarily through gut-homing lymphocyte inhibition and has a distinct systemic immunosuppressive profile.

Mechanistically, these observations are biologically plausible but nuanced. While biologic and targeted therapies—including TNF inhibitors, integrin antagonists, IL-12/23 and IL-23 inhibitors, and JAK inhibitors—attenuate aspects of cell-mediated immunity, they do not uniformly produce the profound T-cell dysfunction associated with disseminated vaccine-strain infections [4,5,6,7,19]. Gut-selective agents such as vedolizumab may have minimal systemic immunosuppressive effects, which may further mitigate risk. At the same time, the theoretical concern remains that impaired cellular immunity could allow the increased replication of attenuated vaccine strains, forming the basis for longstanding guideline caution.

Accordingly, our findings should be interpreted as reassuring but not definitive. While the primary analyses did not demonstrate a statistically significant increase in hospitalizations or emergency department visits in vaccinated patients, the confidence intervals are wide, and rare outcomes were too infrequent to exclude clinically meaningful risk. These findings should be considered exploratory rather than reassuring and should not be used to modify current conservative vaccination guidelines without confirmatory evidence. The absence of a detectable short-term safety signal in this large real-world cohort, together with similar observations across prior studies, suggests that LAV administration during immunomodulatory therapy does not necessarily result in frequent adverse clinical outcomes. However, given the rarity of severe events, wide confidence intervals, and inherent limitations of retrospective Electronic Health Record-based analyses, these data do not establish universal safety. Instead, they support a framework of individualized decision-making.

From a clinical perspective, current guideline recommendations remain appropriate. LAVs should ideally be administered prior to the initiation of biologic or JAK inhibitor therapy, particularly in patients receiving concomitant corticosteroids or other immunosuppressive agents [12,15,19,20]. When vaccination cannot be deferred—such as for yellow fever in unavoidable travel to endemic regions—an individualized risk–benefit assessment is reasonable, incorporating shared decision-making, a review of the immunosuppressive burden, and a close follow-up. Similarly, for nonimmune adults requiring MMR vaccination in high-risk exposure settings, our findings support case-by-case consideration rather than routine administration during therapy.

Overall, our study adds to a growing body of literature suggesting that serious adverse outcomes following LAV exposure in patients with IBD on biologic or targeted therapy are uncommon, particularly when patients are carefully selected. Nevertheless, LAV use in this population should remain individualized and guided by a careful risk–benefit assessment rather than considered routine practice.

## 5. Strengths and Limitations

This study included multiple strengths. Our study includes a large propensity score–matched analysis from multiple centers, allowing for robust findings across diverse healthcare systems and patient populations. The inclusion of multiple immunomodulators across different mechanisms of action enhances generalizability. This study leverages an extensive, multicenter US database, thereby improving external validity and making the cohort more representative of real-world IBD care. Propensity matching achieved an excellent covariate balance, and we used convergent risk and time-to-event analyses across clinically meaningful short-term windows. The study provides real-world evidence in a setting where prospective randomization may be ethically or logistically challenging, offering reassurance that LAVs can be considered in carefully selected patients on immunomodulators.

However, there are also several limitations. TriNetX aggregates de-identified, coded EHR data and returns aggregate outputs; individual chart adjudication is not possible. Exposures, outcomes, and timing therefore reflect coded events and are subject to misclassification. Medication timing is imperfectly captured. Although vaccinations were clustered near biologic initiation to most likely capture those currently receiving immunomodulators, temporary drug holds or dose modifications cannot be excluded. The platform cannot attribute causality, limiting our ability to ascribe outcomes solely to vaccination versus intercurrent illness. Several outcomes, including encephalitis, shingles, and measles—were rare or absent, thereby widening confidence intervals and limiting precision. Further subanalyses were limited by small event counts. Some immunomodulators are newer to the market, and, therefore, fewer patients were available for analysis within these subgroups. Current guidelines generally recommend against administering LAVs to patients actively receiving immunomodulators, which likely limited the overall sample size. Individualized per-biologic and per-vaccine subanalyses (e.g., isolated live zoster or yellow fever vaccination) were underpowered and could not be reliably performed.

A major limitation of this study is the inability to perform drug-class-specific or per-biologic subgroup analyses. Because this study uses de-identified data from the TriNetX platform, the reporting of small cell counts and granular subgroup breakdowns is restricted to protect patient privacy in accordance with HIPAA Safe Harbor standards. The pooled biologic category, therefore, encompasses agents with substantially different systemic immunosuppressive profiles. Future prospective registries or linkage studies with individual-level drug exposure data are needed to address this critical gap.

Another key limitation of this study is the potential for immortal-time bias and healthy-vaccinee bias arising from the asymmetric time-zero definition between cohorts. To improve temporal comparability, the exposure window was restricted such that vaccination occurred within 60 days of biologic initiation; however, vaccinated patients still necessarily survived and remained clinically stable long enough to receive vaccination, which may inherently favor healthier individuals. A rigorous target-trial emulation using biologic initiation as the common time zero and vaccination as a time-varying exposure was not feasible within the current TriNetX analytical framework, which does not support time-varying covariate adjustment or landmark analyses. Accordingly, these findings should be interpreted as observational associations rather than causal estimates.

The timing of vaccination relative to biologic initiation may not reflect the peak immunosuppressive effect, as some patients may have been vaccinated prior to full therapeutic exposure. The specific types of LAVs and individual-level immunization histories were incompletely characterized, limiting the interpretation of the true exposure risk. The variation in immunosuppressive intensity across biologic classes (e.g., gut-selective vs. systemic agents) could not be fully stratified.

Additionally, the baseline immunization status and serologic immunity were not available. Some patients may have received LAVs despite prior immunity (e.g., due to equivocal titers), thereby reducing their true risk of vaccine-related infection and potentially biasing findings toward apparent safety.

## 6. Conclusions

This retrospective propensity-score-matched cohort study did not identify a statistically significant increase in short-term hospitalizations, emergency department visits, fever, rash, encephalitis, or vaccine-related viral infections over six months among patients with inflammatory bowel disease receiving biologic or targeted therapy who were administered live attenuated vaccines compared with unvaccinated controls. However, these findings should be interpreted cautiously and considered exploratory rather than definitive. Important limitations include the retrospective observational design, broad exposure window surrounding biologic initiation, potential time-zero and healthy-vaccinee bias, use of nonspecific clinical outcomes, heterogeneity among included vaccines and biologic therapies, and limited power to evaluate rare vaccine-related adverse events. Consequently, these results should not be interpreted as sufficient evidence to support routine live attenuated vaccine administration after the initiation of immunomodulatory therapy or to alter current conservative vaccination recommendations. Additional prospective studies with more precise exposure timing, biologically specific outcomes, and larger cohorts are needed to further characterize the safety of live attenuated vaccines in this population.

## Figures and Tables

**Table 1:** Baseline characteristics after propensity score matching. IBD subtype and biologic/targeted therapy class included in matching. Percentages for CD and UC do not sum to 100% as patients may carry codes for both conditions, reflecting IBD-unclassified overlap per ICD-10 coding practices in the TriNetX platform.

	Before Matching				After Matching			
Characteristic	Before Matching Biologics w/LAV (n = 672)	Before Matching Biologics w/o LAV (n = 144,165)	*p* Value	SMD	After Matching Biologics w/LAV (n = 672)	After Matching Biologics w/o LAV (n = 672)	*p* Value	SMD
Age at index, mean ± SD	38.8 ± 17.6	38.8 ± 18.8	0.969	0.002	38.8 ± 17.6	39.0 ± 17.8	0.812	0.013
Female sex	73.7%	51.9%	<0.001	0.462	73.7%	73.8%	0.951	0.003
Male sex	26.3%	48.0%	<0.001	0.459	26.3%	26.2%	0.951	0.003
White race	76.2%	76.8%	0.698	0.015	76.2%	76.6%	0.847	0.011
Black or African American	13.5%	9.0%	<0.001	0.143	13.5%	14.0%	0.812	0.013
Asian	4.0%	2.5%	0.009	0.088	4.0%	4.2%	0.890	0.008
Hispanic or Latino	5.5%	5.0%	0.512	0.025	5.5%	5.4%	0.904	0.007
Not Hispanic or Latino	83.8%	73.8%	<0.001	0.245	83.8%	84.4%	0.766	0.016
American Indian/Alaska Native	1.5%	0.4%	<0.001	0.118	1.5%	1.5%	1.000	<0.001
Unknown race	3.7%	7.6%	<0.001	0.168	3.7%	3.3%	0.656	0.024
Other race	2.1%	3.6%	0.036	0.091	2.1%	1.6%	0.545	0.033
Crohn disease	58.9%	38.7%	<0.001	0.414	58.9%	58.9%	1.000	<0.001
Ulcerative colitis	28.9%	23.3%	0.001	0.128	28.9%	29.3%	0.857	0.010
HIV disease	1.5%	0.1%	<0.001	0.161	1.5%	1.5%	1.000	<0.001
Diabetes mellitus	2.4%	3.1%	0.297	0.043	2.4%	3.0%	0.499	0.037
Other specified immunodeficiencies	2.2%	0.7%	<0.001	0.123	2.2%	1.5%	0.313	0.055
Defects in complement system	1.5%	0.1%	<0.001	0.164	1.5%	0.0%	0.002	0.174

**Table 2:** Clinical outcomes from 1 day to 6 months after propensity score matching in patients with inflammatory bowel disease receiving biologic or targeted therapy with and without live attenuated vaccine exposure. Risk ratios (RRs) with 95% confidence intervals (CIs) are shown for each outcome.

**Outcome**	**Biologics with LAV (n = 672)**	**Biologics Without LAV (n = 672)**	**Risk Ratio (95% CI)**	***p* Value**
Inpatient visits	100/672 (14.9%)	103/672 (15.3%)	0.97 (0.75–1.25)	0.819
Emergency department visits	85/672 (12.6%)	76/672 (11.3%)	1.12 (0.84–1.50)	0.450
Fever	24/672 (3.6%)	22/672 (3.3%)	1.09 (0.62–1.93)	0.764
Rash	27/672 (4.0%)	18/672 (2.7%)	1.50 (0.83–2.70)	0.172
Encephalitis	0/672 (0%)	0/672 (0%)	Not estimable	—
Measles	0/672 (0%)	0/672 (0%)	Not estimable	—
Mumps	0/672 (0%)	0/672 (0%)	Not estimable	—
Rubella	0/672 (0%)	0/672 (0%)	Not estimable	—
Varicella	0/672 (0%)	0/672 (0%)	Not estimable	—
Yellow fever	0/672 (0%)	0/672 (0%)	Not estimable	—
**Number of Instances Analysis**				
**Outcome**	**Mean Instances ± SD (LAV)**	**Mean Instances ± SD (No LAV)**		***p* Value**
Inpatient visits	2.97 ± 4.10	2.94 ± 3.10		0.956
Emergency department visits	1.72 ± 1.08	2.45 ± 3.08		0.042
Fever	1.79 ± 2.45	2.09 ± 2.27		0.670
Rash	1.85 ± 1.46	1.61 ± 1.04		0.487

**Table 3:** Clinical outcomes and number of outcome instances from 1 day to 90 days after propensity score matching in patients with inflammatory bowel disease receiving biologic or targeted therapy with and without live attenuated vaccine exposure.

**Outcome**	**Biologics with LAV (n = 672)**	**Biologics Without LAV (n = 672)**	**Risk Ratio (95% CI)**	***p* Value**
Inpatient visits	70/672 (10.4%)	68/672 (10.1%)	1.03 (0.75–1.41)	0.857
Emergency department visits	60/672 (8.9%)	47/672 (7.0%)	1.28 (0.89–1.84)	0.190
Fever	14/672 (2.1%)	14/672 (2.1%)	1.00 (0.48–2.08)	1.000
Measles	0/672 (0%)	0/672 (0%)	Not estimable	—
Mumps	0/672 (0%)	0/672 (0%)	Not estimable	—
Rubella	0/672 (0%)	0/672 (0%)	Not estimable	—
Varicella	0/672 (0%)	0/672 (0%)	Not estimable	—
Yellow fever	0/672 (0%)	0/672 (0%)	Not estimable	—
**Number of Instances Analysis**				
**Outcome**		**Mean Instances ± SD (LAV)**	**Mean Instances ± SD (No LAV)**	***p* Value**
Inpatient visits		2.04 ± 2.30	2.82 ± 2.90	0.082
Emergency department visits		1.52 ± 0.91	2.17 ± 2.38	0.053
Fever		1.93 ± 2.65	2.36 ± 2.65	0.672

## Data Availability

The data that support the findings of this study were obtained from the TriNetX Research Network, a federated, de-identified electronic health record database Accessed on 15 May 2026 (https://trinetx.com). Due to data use agreements, the raw data is not publicly available.
